# Case Report: Phenotypic and genetic characterization of a presumptive sporadic hypothalamic hamartoma in a standard Schnauzer dog

**DOI:** 10.3389/fvets.2025.1591863

**Published:** 2025-05-27

**Authors:** Theofanis Liatis, Elizabeth Attree, Laura Ruiz De Alejos Blanco, Patrick Santens, Alberta De Stefani, Androniki Psifidi

**Affiliations:** ^1^Department of Clinical Science and Services, Royal Veterinary College, University of London, Hatfield, United Kingdom; ^2^Department of Pathobiology and Population Sciences, Royal Veterinary College, University of London, Hatfield, United Kingdom; ^3^Department of Neurology, Universitair Ziekenhuis Ghent, Ghent, Belgium

**Keywords:** brain heterotopia, sonic hedgehog pathway, *SEPTIN8* gene, *UBXN10* gene, *BLOC1S1* gene, repetitive movements, ciliopathy, cilia

## Abstract

**Introduction:**

Hypothalamic hamartoma (HH) is a rare suprasellar developmental lesion that resembles ectopically located grey matter within the hypothalamus. Genetic mutations in genes involved in the sonic hedgehog intracellular pathway have been reported in humans with HH. Hypothalamic hamartoma has been reported in dogs; however, no genetic mutation has been associated with it. The aim of this study was to phenotypically and genetically characterize presumptive sporadic HH in a dog.

**Case presentation:**

A 7-month-old male Schnauzer was presented with chronic left head tilt, episodes of imbalance, circling to the left, and continuous repetitive movements of the head and neck to the left. These episodes could increase with stress or interaction, and the dog could not be distracted out of it. Clinical examination was normal. Neurological examination revealed a continuous repetitive involuntary movement of the head and neck to the left and left ocular torsion. Haematology and serum biochemistry were within normal limits. Magnetic resonance imaging of the head raised a suspicion of HH. Whole genome sequencing and genetic analysis identified germline variants with a high predicted impact on the encoding proteins in the candidate genes *SEPTIN8*, *UBXN10*, and *BLOC1S1*, which are involved in ciliogenesis and may be associated with the manifestation of HH in this dog.

**Conclusion:**

Sporadic HH should be considered in the differential diagnoses list for a young dog with chronic progressive intracranial neurologic signs and may be genetically associated with germline mutations in primary cilia-related genes.

## 1 Introduction

In humans, hypothalamic hamartoma (HH) is a rare suprasellar developmental non-neoplastic lesion in which ectopically located brain tissue can be found within the ventral hypothalamus (1, 2). This tissue resembles grey matter and contains varying proportions of neurons, glia, and fibre bundles (1). In humans, there are two clinical phenotypes of HH: the intrahypothalamic, located within the hypothalamus, and the parahypothalamic, located in the third ventricle (2). Both have been associated with a typical clinical semiology; specifically, intrahypothalamic HH has been associated with pharmacoresistant epilepsy, gelastic seizures (i.e., uncontrolled laughing) as its hallmark, developmental regression, psychiatric, behavioural comorbidities, and precocious puberty. Parahypothalamic HH has only been associated with endocrinopathy (e.g., precocious puberty) (2). Causative genetic mutations, mostly located in the sonic hedgehog (SHH) pathway, have been found in 50% of nonsyndromic HH in humans (3).

A canine hypothalamic hamartoma was first reported in 1977 (4), and since then it has been reported in three more dogs either as a localized entity or as hamartoma extending to the thalamus (5–7). In dogs, neurological signs were variable: they ranged from paroxysmal ataxia, disorientation, cataplexy, circling, absence of interaction with the owners, and sleep deprivation to head tilt, pacing, circling, hypermetria, opisthotonus, nystagmus, positional strabismus, and postural reaction deficits (4, 6, 7). To date, genetic studies for HH have not been reported in the veterinary literature. Herein we describe the clinical, magnetic resonance imaging (MRI), and genetic findings of a dog with presumptive HH.

## 2 Case presentation

### 2.1 Signalment and history

A 7-month-old male entire standard Schnauzer was presented to the hospital with a history of chronic progressive left head tilt, episodes of imbalance, compulsive movements of the head and neck, and occasional left circling. Further investigations were performed with owner consent.

### 2.2 Clinical and neurological findings

Clinical examination was unremarkable overall, though the right ear seemed pulled to the left. Subjectively the right cervicoauricularis muscle was considered hypertonic upon palpation.

Neurological examination revealed an inappropriate mentation with the dog being scared, over-reactive, and abrupt in any interactions. Posture analysis revealed a left head tilt and a constant, repetitive, non-rhythmical rotation of the head and neck to the left, causing a simultaneous left rotation of the right ear pinna along with the rotation of the head (Video). Gait analysis revealed occasional compulsive tight circling to the left, but not overt ataxia or paresis. Postural reactions and spinal reflexes were intact in all limbs. Cranial nerve assessment revealed a left ventral positional strabismus. During the head rotation movement, an ocular torsion of the left eye, simultaneous with the head movement, was evident only in the eye ipsilateral to the rotation of the head. Palpation of the vertebral column did not reveal spinal hyperaesthesia. Neuroanatomical localisation was consistent with multifocal brain disease, including the forebrain and brainstem.

### 2.3 Diagnostic investigation findings

**Figure 1:**
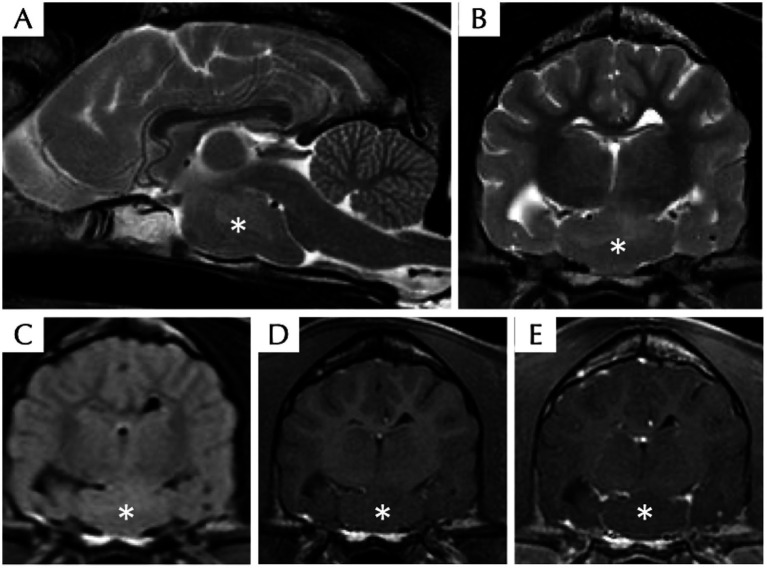
Magnetic resonance imaging of the head of a dog with suspected hypothalamic hamartoma, including T2W sagittal **(A)**, T2W transverse **(B)**, T2 FLAIR transverse **(C)**, T1W pre- **(D)**, and post- **(E)** contrast transverse sequences. There is a well-defined ovoid T2W, T1W, and FLAIR isointense compared to grey matter intra-axial mass (asterisk) without contrast enhancement at the level of the ventral middle cranial fossa extending along the cranial base within the region of hypothalamus.

The venous blood gas analysis, haematology, serum biochemistry, bile acid stimulation test, and C-reactive protein were within normal limits. Radiography of the thorax and abdomen were unremarkable. Magnetic resonance imaging of the head revealed a well-defined ovoid suprasellar mass (suspected intra-axial) without contrast enhancement at the level of the middle cranial fossa extending along the cranial base. The lesion was iso-intense to grey matter on T1W, T2W, and FLAIR images (Figure 1). The lesion appeared to be in broad contact with the left ventral margin of the thalamus (hypothalamus). Mass effect, displacement of the pituitary gland cranially with mild compression to the thalamus and midbrain dorsally, and midline shift to the right were present. There were a few regions of mildly heterogeneously T2W and FLAIR hyperintensity compared to the white matter surrounding the lesion, which is likely due to secondary oedema resulting from the compression. Cerebrospinal fluid tap was not attempted, and electroencephalography was declined by the owners. The presumptive diagnosis of HH was based on clinical presentation and characteristic MRI features, whereas neoplasia or inflammatory disease were considered unlikely. Due to the suspected HH diagnosis, approximately 2 mL of EDTA whole blood was also collected by venipuncture for DNA extraction to send for whole genome sequencing with informed consent from the owner, to investigate for possible germline mutations associated with this developmental disorder.

### 2.4 Treatment and outcome

Treatment trial for suspected movement or behavioural disorder with fluoxetine was initiated at 1.5 mg/kg PO q24h for 10 days, but it did not alter the frequency or severity of the episodes, and it was discontinued due to side effects (lethargy, anorexia). An antiseizure drug trial treatment with levetiracetam 30 mg/kg PO q8h was attempted for 1 month; once again, there was no change in the episode frequency or severity, and this drug was also discontinued. During a follow-up call 1 year later, the dog remained drug-free, and the clinical status was unchanged, with persistent episodes of circling and repetitive head and neck movements.

### 2.5 Whole genome sequencing analysis

Details of blood DNA extraction, whole genome sequencing (WGS), and functional annotation of the identified genetic variants in potential candidate genes are described (Supplementary material S1). Briefly, genomic DNA was extracted from blood using the DNeasy blood and tissue kit (Qiagen, Hilden, Germany) with a few modifications to study germline mutations potentially associated with this developmental condition. WGS analysis and variant calling were performed using the GATK v4.1.6 (8) best practice workflow and the canis_lupus_familiaris_ROS_Cfam_1.0 reference genome. Variants were filtered out using the Dog10K project (9) data to identify only unique variants to this animal, as a rare (unique) genetic variant should be responsible for this rare condition.

**Figure 2:**
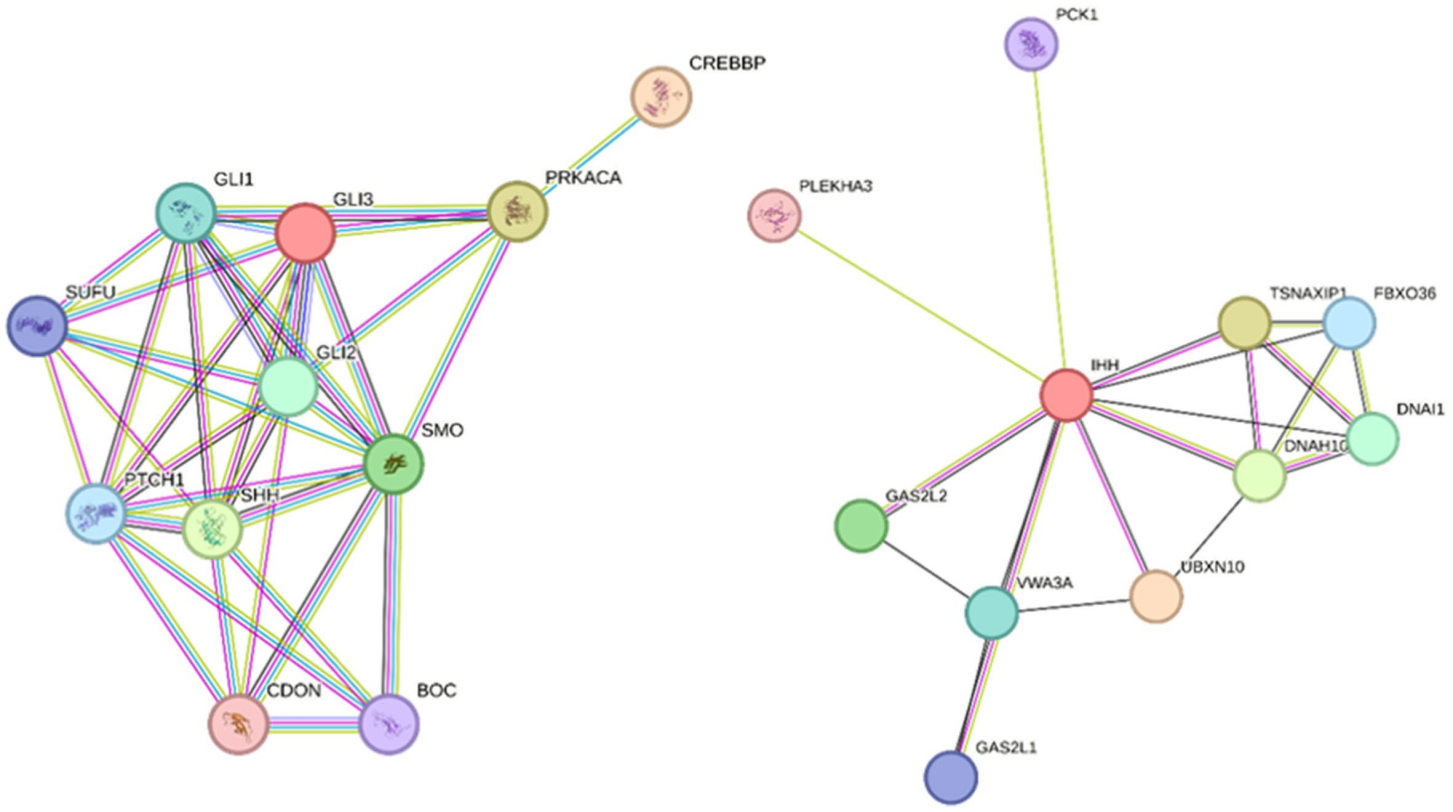
String analyses to identify pathways of interest with genes interacting with candidate HH genes according to the human literature. Pathway 1 (left) input genes: PRKACA, SHH, IHH, SMO, CREBBP, GLI2, and GLI3; string parameters: high confidence 0.7, no more than 5 interactions. Pathway 2 (right) input gene: IHH, string parameters; medium confidence 0.4, no more than 10 interactions.

The focus of the WGS data analysis was on 27 potential candidate genes based on the human HH literature (10) (*PRKACA, SHH, IHH, SMO, CREBBP, GLI2,* and *GLI3*) and further pathway analysis using STRING v12.0 (11) to identify other interacting genes involved in the same key neurological pathways (Table 1; Figure 2). In addition, a search across the genome for genetic variants with a predicted high impact located within genes related to ciliogenesis was performed. The genetic variants identified within these 27 candidate genes were extracted from the WGS data for further interrogation. Analysis of the genomic regions of interest included +10 kb flanking regions to identify genetic variants potentially located in regulatory regions controlling the expression of these candidate genes. In total, 7,805 genetic variants identified across the genomic regions of interest were annotated using the variant effect prediction (VEP) tool from Ensembl (12). After removal of 629 intergenic and 6,927 intronic variants, 249 remained, and these were filtered for uniqueness against the Dog10K project variants (9). Thus, 118 unique variants remained (1-high impact, 24-missense, and 93 modifier impact variants) for further analysis (Supplementary material S2 and S3). From these unique genetic variants, the high-impact one was located in *SEPTIN8*; from the missense ones, only one had a low SIFT score, suggesting a deleterious effect in the encoded protein. The unique high-impact variant (homozygous alternative C/T) in *SEPTIN8* was a splice acceptor variant consequence. Variants in splice regions can significantly affect the complex splicing mechanism, impacting the mRNA, for example, from exon loss or intron gain, either partial or complete, with downstream effects on the resulting protein sequence (13–15). Septin proteins have been previously linked to a number of neurological diseases, including frontotemporal lobar degeneration, Alzheimer’s disease, or Huntington’s disease (16).

**Table 1:** The genes used to select the genomic windows for variant effect prediction based on existing literature and SHH and IHH pathways.

Gene stable ID	Gene name	Chromosome	Gene start-end (bp)	Window start-end (bp)
*ENSCAFG00845000394*	*KIF7*	3	53,046,492–53,065,700	53,036,492–53,075,700
*ENSCAFG00845001422*	*FBXW11*	4	40,613,835–40,738,782	40,603,835–40,748,782
*ENSCAFG00845001617*	*CREBBP*	6	37,649,589–37,750,377	37,639,589–37,760,377
*ENSCAFG00845001925*	*PRKACB*	6	64,155,572–64,269,867	64,145,572–64,279,867
*ENSCAFG00845002733*	*VWA3A*	6	23,612,471–23,671,881	23,602,471–23,681,881
*ENSCAFG00845003000*	*UBXN10*	2	79,240,192–79,246,641	79,230,192–79,256,641
*ENSCAFG00845003905*	*PTCH1*	1	71,542,581–71,613,625	71,532,581–71,623,625
*ENSCAFG00845006665*	*TSNAXIP1*	5	82,082,255–82,096,914	82,072,255–82,106,914
*ENSCAFG00845009497*	*GAS2L2*	9	38,729,912–38,737,587	38,719,912–38,747,587
*ENSCAFG00845009814*	*CDON*	5	8,391,716–8,489,974	8,381,716–8,499,974
*ENSCAFG00845010518*	*SPOPL*	19	42,608,284–42,674,020	42,598,284–42,684,020
*ENSCAFG00845011039*	*KIF3A*	11	21,774,475–21,830,123	21,764,475–21,840,123
*ENSCAFG00845011227*	*SPOP*	9	26,480,865–26,548,177	26,470,865–26,558,177
*ENSCAFG00845011836*	*GLI1*	10	1,573,578–1,585,034	1,563,578–1,595,034
*ENSCAFG00845013237*	*SHH*	16	20,382,707–20,392,037	20,372,707–20,402,037
*ENSCAFG00845018240*	*GLI3*	18	7,846,935–8,116,066	7,836,935–8,126,066
*ENSCAFG00845020029*	*FBXO36*	25	42,461,727–42,558,431	42,451,727–42,568,431
*ENSCAFG00845020497*	*BTRC*	28	14,456,356–14,641,377	14,446,356–14,651,377
*ENSCAFG00845020728*	*SMO*	14	7,284,738–7,307,899	7,274,738–7,317,899
*ENSCAFG00845022559*	*PLEKHA3*	36	22,247,913–22,273,936	22,237,913–22,283,936
*ENSCAFG00845023753*	*DNAI1*	11	52,086,129–52,212,664	52,076,129–52,222,664
*ENSCAFG00845024723*	*PRKACA*	20	48,926,580–48,944,481	48,916,580–48,954,481
*ENSCAFG00845025024*	*SUFU*	28	15,466,996–15,589,728	15,456,996–15,599,728
*ENSCAFG00845026918*	*GLI2*	19	30,501,230–30,755,556	30,491,230–30,765,556
*ENSCAFG00845028975*	*IHH*	37	25,633,157–25,638,407	25,623,157–25,648,407
*ENSCAFG00845029845*	*BOC*	33	17,801,336–17,876,142	17,791,336–17,886,142
*ENSCAFG00845029983*	*PCK1*	24	43,403,838–43,409,160	43,393,838–43,419,160

The only missense mutation (homozygous G/T) with a deleterious SIFT score in the genomic regions of interest unique to this animal was UBX Domain Protein 10 (*UBXN10*), located in and involved in ciliogenesis (17). The mutation identified was predicted to cause an amino acid change from alanine (A) to aspartate (D) in the fifth amino acid of the protein, which can impact 3D protein structure and functional efficiency. The SIFT score assigned to this protein (0.01) is also predicted to affect protein function (18, 19).

The modifier variants located in 5 ‘untranslated regulatory regions (UTR) of these 27 candidate genes were investigated for an effect on the promoter region using the Softberry FPROM software, but the analysis did not prioritise any of these 93 variants.

Expansion into the investigation of unique high impact variants across the whole genome of this animal identified 3,679 such variants; 2,902 of these were not present in Ensemble, 5 of them showed deleterious SIFT scores, with a single start loss variant in the *BLOC1S1* gene having a confident deleterious SIFT score of 0.01; *BLOC1S1*, a component of the BLOC1 protein, is involved in endosomal and lysosomal trafficking, particularly in the biogenesis of lysosome-related organelles and indirectly to endosome-primary cilia pathway (20–22), and it has been previously linked to neurite extension (20), hypomyelination, seizure, epilepsy and dystonia in humans (22).

## 3 Discussion

This is the first case combining phenotypical and genetic characterization of suspected HH in a dog. Hypothalamic hamartomas are rare, non-neoplastic heterotopic developmental lesions with important underlying genetic causative factors (23) that develop in a region called the tuber cinereum, which is a part of the hypothalamus located between the mamillary bodies and the optic chiasm (2). At least 37% of humans with HH had genetic causative variants in the sonic hedgehog (SHH) pathway (3); which regulates neurogenesis in the central nervous system, specifically cell patterning in early hypothalamic development (2, 24). Approximately 95% of HH cases are non-syndromic or sporadic. HH is the sole clinical entity where the associated mutation is not inherited from parents (2, 11, 25); the remaining 5% can be syndromic, associated with other developmental abnormalities, such as Oro-Facial Digital Type VI syndrome or Pallister-Hall syndrome (26). As the current dog case manifested with vestibular signs and did not have any other obvious concurrent developmental abnormalities, a nonsyndromic HH was suspected.

In humans, the syndromic HH has been associated with genetic mutations in the *GLI3* gene (2, 10, 25) and other genes related to cilia function. The GLI3 is a transcriptional activator and repressor of downstream SHH pathway targets. The hedgehog family of proteins comprises SHH, Indian hedgehog (IHH), and desert hedgehog, which are versatile signalling molecules involved in a wide spectrum of biological events, including cell differentiation, proliferation, and survival, and they play critical roles from embryogenesis to adult stages (24). The SHH pathway proteins localize to the ubiquitous cell organelle, primary cilia, to facilitate cell-to-cell interactions via signal transduction pathways during development (26). The SHH pathway depends upon a cellular organelle, the primary cilia, to effectively transduce SHH signalling during development (26). Primary cilia are single non-motile axonemes that extend out of the cell membrane of most mammalian cells, including neurons, and function like a cellular antenna to regulate cell-to-cell interactions and signalling during development (26). Primary cilia play an important role in SHH signalling as well as being implicated in neurogenesis, migration, and axon guidance (27). Disruption of cilia genes, “ciliopathies,” leads to altered developmental SHH signalling responses (26) and, in severe cases, to forebrain malformations (28). Interestingly, primary cilia are present within the developing mouse hypothalamus on the neural progenitor cells and committed neurons, where they project 1–2 μm in length from the cell surface (28). A genetic study of human sporadic HH found multiple genetic mutations in genes involved in the SHH pathway, involving genes encoding proteins that localise to the primary cilia (23), and HH is thus a proposed “ciliopathy” (26).

Although we did not identify a variant of interest in the *GL13* gene of this dog case, two significant variants of interest were identified in potential candidate genes related with cilia function which were unique upon comparison to the variants from the dog reference genome and the Dog10K project data, which includes genetic variants from approximately 2000 individual dogs (9). The first was the highly predicted impact splice acceptor variant in *SEPTIN8* (29–32), which codes for sept8 septin. Septins are a family of GTP-binding proteins that associate with cellular membranes and the cytoskeleton and specifically contribute to the regulation of numerous cell processes, such as cytokinesis (33). Sept8 is situated at the base of the cilia. (33) Septin depletion *in vivo* produces phenotypes implicating nodal, motile, and primary cilia (33). Therefore, septins are involved in ciliogenesis, though the specific way remains unknown (33).

The other significant variant was a deleterious mutation predicted to affect protein function in *UBXN10*, which has also been previously implicated in ciliogenesis, as depletion of *UBXN10* in zebrafish embryos leads to left–right asymmetry defects (17). Moreover, another unique high-impact variant was identified in *BLOC1S1,* which, although not a ciliary gene, has been shown to play an indirect role in ciliary assembly (34), and it might therefore may also play a role here.

Our case was diagnosed with HH as the most likely diagnosis. This is the second case report describing MRI features of a suspected HH in a dog. In the previously reported case in a Vizsla, the MRI lesions were mildly T2W hyperintense (7); in our case, the lesion was mainly T2W isointense with few T2W hyperintense regions. Differential diagnoses for this suprasellar lesion included HH, glioma, rare embryonal neoplasia (e.g., craniopharyngioma), or, less likely, pituitary gland adenoma or adenocarcinoma (7). The fact that, in our case, there is a lack of contrast enhancement in the MRI, the age of the dog, the location of the lesion, the signalment, and clinical presentation, was suggestive of HH as the most likely diagnosis. Following Delalande’s classification system in humans (2), our case was considered to have a type III HH, differing from the previously published case, which had a type IV (7). Although the lesion does contact the left ventral margin of the thalamus/hypothalamus, most of the mass is situated in the sellar/suprasellar region and is bilaterally symmetrical; deciding whether the mass originates by the left hypothalamus based solely on imaging features is thus no more than a hypothesis, and it warrants further histological assessment. Our case was considered a sporadic case of HH based on the (a) lack of evidence of other congenital abnormality upon examination, (b) lack of familial history in the extended family tree provided, and (c) lack of identification of an exonic genetic variant with high impact in *GLI3* (aside from a single 5 prime UTR variant in this gene).

Our case manifested a very discrete and unusual continuous repetitive involuntary movement of the head and neck to the left, during which the dog appeared conscious. During this episode, ocular torsion of the left eye towards the direction of the head movement was evident. The clinical hallmark of HH in humans is epileptic seizures, specifically gelastic seizures (1, 4). These are not recognised in animals. Although electroencephalography was not performed in this case, an epileptic origin of the repetitive movements was considered unlikely due to the present consciousness of the dog, the lack of autonomic and post-ictal signs, their continuous nature, and the lack of response to the antiseizure drugs (levetiracetam). An obsessive-compulsive disorder was considered less likely as there was no response to behavioural drugs (fluoxetine) and the dog could not be distracted out of it. A movement disorder was considered less likely given the continuous nature of the events. A continuous vestibular paroxysm was considered possible given the other accompanying neurological signs (circling, occasional ataxia, head tilt) and the fact that the episodes were becoming more intense with a changing environment or upon excitement. Episodic ocular torsion and skew deviation (towards the side of the lesion) have been seen in humans with mesodiencephalic lesions (35). In these patients, there is an initial stage where a torsional fast eye movement takes place, during which rotatory nystagmus can be present, and a second phase in which cessation of the ocular torsion and skew deviation occurs (35). In one human case, contralateral dystonic movements of the limbs were also evident (35). Rotatory nystagmus has been reported in humans with HH and it has been attributed to mass effect and secondary compression to the midbrain and more specifically the interstitial nucleus of medial longitudinal fasciculus and the interstitial nucleus of Cajal (36). In our case, the HH was likely arising from the left hypothalamus, and thus there was more compression at the left than the right side of the brain. Additionally, the HH was extending caudally, causing direct compression to the midbrain. However, although the type and location of the HH and the presence of ocular torsion in our dog during the episode could indicate a vestibular origin, the repetitive movement of the head and neck and the tonicity of the contralateral ear were not typical for a vestibular paroxysm. A movement disorder (e.g., myoclonus) was considered another potential explanation. Movement disorders (i.e., choreoballistic movements) have been reported only in a case report and were attributed to secondary compression of the basal nuclei (37). However, in our dog, we considered myoclonus as a potential explanation. Reticular myoclonus manifesting as jerks occurring at random moments throughout the day and interfering with activities of daily living, such as eating and drinking, without auditory or sensory triggers, has been described in humans with brainstem lesions (38). The hallmark of brainstem myoclonus is an initial muscle activation in muscles innervated in the lower brainstem (sternocleidmastoid or trapezius muscles) followed by a sequence of both higher brainstem innervated muscles and muscles innervated further down the spinal cord (38). On the other hand, this movement could also represent a negative myoclonus (asterixis) (39). Asterixis has been associated usually with hepatic encephalopathy; however, unilateral asterixis has specifically been associated with unilateral brainstem lesions (39). In this case, a short interruption of activity in muscles sustaining the normal head position against gravity could lead to short hypotonia of one side and elicit the abnormal movement towards one side (negative myoclonus equivalent).

Nearly 50% of HH cases in humans are attributed to somatic genetic variants implicated in the SHH signalling pathway; however, a more recent concept suggests that HH may arise from germline and somatic biallelic mutations in cilia, including genes coupled to the SHH route (40). Somatic mutations were beyond the scope of this study since this HH was not considered a neoplastic process and access to the affected tissue was not possible. Nevertheless, we cannot exclude the possibility of somatic mutations playing a role in HH in dogs.

Limitations of this case report included the lack of a final diagnosis confirmation, as the dog remained alive at the time of writing. Additionally, the owner declined surgical biopsy; a brain biopsy was therefore not done. Another limitation included the lack of electroencephalography to define the nature of the involuntary movements. Concurrent developmental abnormalities were ruled out and a nonsyndromic HH was suspected based solely on clinical examination, blood tests, and thoracic and abdominal radiographs; however, any clinically silent developmental abnormalities could not be completely ruled out based on these assessments only. Cerebrospinal fluid analysis or endocrinological tests were not performed.

In conclusion, this is the first report of whole genome sequencing analysis in a dog with presumptive sporadic HH. This study revealed unique to this case genetic variants of deleterious predicted effect in three genes, *SEPTIN8*, *UBXN10,* and *BLOC1S1,* associated with neurological development and function, particularly in cilia and ciliogenesis. Moreover, an unusual continuous repetitive involuntary movement of the head and neck was associated with HH in this case. These movements were attributed to compression of the midbrain. Hypothalamic hamartoma should therefore be included in the differential diagnosis list in a dog with involuntary movements. Further genetic studies are needed to validate these results as well as study the potential role of somatic mutations to unravel further the genetic background of this rare disease in histopathologically confirmed cases.

## Data Availability

The datasets presented in this study can be found in online repositories. The names of the repository/repositories and accession number(s) can be found in the article/Supplementary material.
