# Supplementary material for: Case Report: Phenotypic and genetic characterization of a presumptive sporadic hypothalamic hamartoma in a standard Schnauzer dog
Source: Front Vet Sci. 2025 May 27;12:1591863. doi: 10.3389/fvets.2025.1591863 (PMC12150237; doi:10.3389/fvets.2025.1591863)
Supplement: Supplementary file 1 [file Data_Sheet_1.docx]

**SUPPLEMENTARY MATERIAL S1**

**Supplementary Methods**

**DNA extraction and sequencing**

Genomic DNA was extracted from blood using a commercial kit, DNeasy blood and tissue kit (Qiagen, Hilden, Germany), following the manufacturers’ instructions with a few modifications. Briefly, in duplicate 50µL of anticoagulated blood was mixed with 50µL phosphate buffered saline (PBS), this was mixed with 40 µL proteinase K, 20 µL RNase and 80 µL PBS to a final volume of 240 µL. After following manufacturer instructions for addition of buffer AL and incubation, 300 µL of 100% ethanol was added to the sample, mixed by vortex and incubated at room temperature for 30 minutes. After centrifugation, following manufacturer instructions, flow through was discarded and 300 µL Buffer AW1 added and incubated for one minute at room temperature before centrifugation and discarding of flow through. The addition of buffer AW1, incubation and centrifugation were repeated three times before addition of 300 µL Buffer AW2. This was incubated for one minute at room temperature before centrifugation and discarding of flow through. Addition of Buffer AW2, incubation, and centrifugation was repeated three times. The remaining steps were conducted following the manufacturer’s instructions and the final yield of genomic DNA eluted in 50 µL Buffer AE. Extracted DNA quality and concentration were assessed using Qubit™ (dsDNA BR Assay Kit, Invitrogen, Massachusetts, USA) according to manufacturer’s instructions.

Sample quality control was performed using Agilent 5400 Fragment Analyzer System, while library preparation and whole genome sequencing (WGS) was outsourced to Novogene Ltd., Cambridge, UK. Whole genome sequencing analysis and variants were called following the GATK (v4.1.6) best practice workflow (Van der Auwera and O'Connor, 2020)(Van der Auwera and O'Connor, 2020). Quality control of the sequencing reads and trimming to improve read quality was performed by FASTQC (v0.11.9) and Trimmomatic (v0.39) software’s, respectively (default parameters). Alignment of reads to the reference genome (canis_lupus_familiaris_ROS_Cfam_1.0) was performed using BWA-MEM algorithm (v0.7.10) (Li, 2013) (Li, 2013).

**STRING analysis**

Specific genes of interest with respect to HH were identified from literature (*PRKACA, SHH, IHH, SMO, CREBBP, GLI2* and *GLI3*). Using these genes, STRING (v12.0) pathway analysis was used to identify further genes of potential interest that interact strongly with the seven candidate genes in relevant pathways and therefore potentially involved in HH. Parameters for the STRING pathway analysis for *PRKACA, SHH, SMO, CREBBP, GLI2* and *GLI3* of the aforementioned genes (*all but* IHH) were: high confidence 0.7, no more than 5 interactions. For the STRING pathway analysis of *IHH* the parameters were medium confidence 0.4, no more than 10 interactions. Genes in pathways with those listed above, identified by STRING (v12.0) pathway analysis yielded the gene list detailed in Table 1 and Figure 2.

**Variant Effect Prediction analysis**

Whole genome genetic variants were annotated by the ensemble tool VEP^15^ (v110) to identify genetic variants of interest with a predicted high, moderate or modifier impact in the encoded protein. The genomic windows of the genes in Table 1 were extracted using ensemble Biomart (with +10kb flanking either side) to generate the genomic regions of interest. Hard filtering was applied to retain only variants with a predicted high impact (across the whole genome) and all variants of any impact within the genomic regions of interest (Table 1). Further filtering was applied to remove intronic and intergenic variants.

The retained variants were compared to the variants identified from the 1987 canine individuals in the Dog10K project (PRJEB62420) (Meadows et al., 2023). Unique variants were identified using bedtools intersect (Quinlan and Hall, 2010). Whole genome high impact variants were further filtered for confident deleterious score and the absence of an existing identified variant.

**Softberry analysis**

The genetic variants with a potential modifier impact identified in 5 prime untranslated regulatory regions (UTR) may affect the promoter of a genes and thus it’s gene expression. In order to identify such variants promoter prediction analysis was performed using the Softberry algorithm (Softberry FPROM, [www.softberry.com](http://www.softberry.com), default parameters). The sites of predicted promotors on the reference sequence for genes (+/- 10kb) with such variants was compared to the alternative sequences (+/- 10kb) generated by the whole genome sequence analysis.

**REFERENCES**

Van Der Auwera GA, O’ Connor BD. Genomics in the cloud: using Docker, GATK, and WDL in Terra. O'Reilly Media. 2020

Andrews S. FastQC: a quality control tool for high throughput sequence data (2010). 2017.

Bolger AM, Lohse M, Usadel B. Trimmomatic: a flexible trimmer for Illumina sequence data. *Bioinformatics* 2014; 30**,** 2114-2120.

Li H. Aligning sequence reads, clone sequences and assembly contigs with BWA-MEM. *arXiv preprint arXiv* 2013; 1303.3997.

Meadows JR, Kidd JM, Wange GD, et al. Genome sequencing of 2000 canids by the Dog10K consortium advances the understanding of demography, genome function and architecture. *Genome biology* 2023; 24: 187.

Quinlan AR, Hall IM. BEDTools: a flexible suite of utilities for comparing genomic features. *Bioinformatics* 2010; 26: 841-842.

Van Der Auwera GA, O’Connor BD. Genomics in the cloud: using Docker, GATK, and WDL in Terra, O'Reilly Media, 2020.
