# Supplementary material for: Case Report: Phenotypic and genetic characterization of a presumptive sporadic hypothalamic hamartoma in a standard Schnauzer dog
Source: Front Vet Sci. 2025 May 27;12:1591863. doi: 10.3389/fvets.2025.1591863 (PMC12150237; doi:10.3389/fvets.2025.1591863)
Supplement: Supplementary file 3 [file Data_Sheet_3.docx]

**Supplementary Material S3**

**DNA variants identification**

An initial list of seven genes of interest (i.e. *PRKACA, SHH, IHH, SMO, CREBBP, GLI2* and *GLI3*) was created based on the literature in the SHH pathway.^12^ This list was used in a STRING (v12.0)^13^ pathway analysis to identify further genes of potential interest that interact strongly with the seven candidate genes in relevant pathways and therefore could play a potential role in HH. Parameters for the STRING pathway analysis for six of the aforementioned genes (*all but* IHH) were: high confidence 0.7, no more than 5 interactions. For the STRING pathway analysis of *IHH* the parameters were medium confidence 0.4, no more than 10 interactions. Genes in pathways with those listed above, identified by STRING (v12.0) pathway analysis^13^ yielded the gene list detailed in Table 1 and Figure 2.

Genomic locations for the genes of interest and pathway interactors were identified using ensemble Biomart.^14^ The sequencing of these genes as well as 10kb genomic regions either side of the specified gene window were extracted from the whole genome sequencing data for further downstream analysis.

The identified genetic variants in the genomic regions of interest (Table 1) were annotated by the ensemble tool variant effect prediction (VEP)^15^ analysis (v110) in order to identify genetic variants of interest with a predicted high, moderate or modifier impact in the encoded protein.

The genetic variants with a potential modifier impact identified in 5 prime untranslated regulatory regions (UTR) may affect the promoter of a genes and thus it’s gene expression. In order to identify such variants promoter prediction analysis was performed using the Softberry algorithm (Softberry FPROM, www.softberry.com). The sites of predicted promotors on the reference sequence for genes (+/- 10kb) with such variants was compared to the alternative sequences (+/- 10kb) generated by the whole genome sequence analysis.

A total of 7805 genetic variants identified across the genomic regions of interest (Table 1). The assigned consequences were 7706-modifier, 71-low, 26-moderate and 2-high. Removal of 629 intergenic and 6927 intron variants reduced the number of modifier variants to 175. After comparison of variants to those identified in the Dog10K project 118 unique variants remained (1-high, 24-moderate and 93-modifier impact). The number and type of identified SNP for each of the 61 genes analysed is detailed in Table 3. For the primary genes of interest (*PRKACA, SHH, IHH, SMO, CREBBP, GLI2* and *GLI3*), only intron variants were identified in *SHH* and *IHH.* After comparison of variants to those identified in the Dog10K project, unique non-intronic/intergenic variants were identified for: *PRKACA* (3 x 3 prime UTR), *SMO* (1 x 3 prime UTR) and *GLI2* (4 x 3 prime UTR, 5 x missense). Of the additional genes in Table 2 all of the SNPs identified in; *FBXW11, TSNAXIP1, PLA2G2C, NUTF2, CENPT, RANBP10, INHBE, TRIP12* and *ACTR1A* were intron variants.

Two high impact variants were identified in *GAS2L2* and *SEPTIN8* and resulted in a frameshift, splice region variant and a splice acceptor variant, respectively. The high impact variant identified in *GAS2L2* (the only non-intron variant identified in this gene) was found to be in the coding sequence and cause an amino acid change. This change (deletion of C nucleotide) results in a shift of the stop codon downstream by one codon and the addition of another Arginine (R), displayed in Figure 3. Addition of an amino acid to a protein can have an impact on the folding of the protein, therefore, the 3D structure and downstream effects on a protein biding infinity or efficiency.

Identified non-intergenic and non-intronic variants assigned modifier, moderate and high impact are listed in supplementary table S1. Mutations with a predicted moderate impact on the encoded protein were all missense variants located in coding regions of the genes *KIF7* (tolerated 0.38)*, VWA3A* (tolerated 0.11-1)*, UBXN10* (deleterious 0.01)*, CDON* (tolerated 0.17-0.58)*, GLI2* (tolerated 0.26-1)*, CCNI2* (tolerated low confidence 0.74-1)*, INHBC* (tolerated 0.2)*, GLI1* (tolerated 0.28-0.29)*, ASF1B* (tolerated low confidence 0.3) and *BOC* (no SIFT score). Modifier consequence variants in the 5 prime UTR regulatory regions were identified for *PRKACB, PTCH1, SPOPL, DNAI1, CNTFR, GLI1, ARHGAP9, GLI3, TRIM8, AHCYL2, FKBP7, PRKACA, C19orf67* and *BOC*. In-frame insertions with a predicted moderate impact were identified in genes: *C17orf50* and *CNTFR*. The only missense mutation with a deleterious SIFT score was UBX Domain Protein 10 (*UBXN10*), located in and involved in ciliogenesis.^16^ The mutation identified was predicted to cause an amino acid change from alanine (A) to aspartate (D) in the fifth amino acid of the protein which, as mentioned previously can impact 3D protein structure and functional efficiency. The SIFT score assigned to this protein (0.01) is also predicted to affect protein function.^17,18^

High impact variants across the genome were also identified by VEP and filtered for uniqueness compared to other canine genomes, unique variant not previously identified and confident deleterious SIFT score. This resulted in one high impact variant (homozygous start lost) in *BLOC1S1*.

The Softberry analysis, was performed for six (*PRKACB, CNTFR, ARHGAP9, AHCYL2, FKBP7* and *BOC*) genes with variants identified in 5 prime untranslated regulatory regions (UTR), Genes with altered predicted promoter sites compared to the reference sequence were *PRKACB, CNTFR, ARHGAP9, AHCYL2, FKBP7* and *BOC*. Six variants were identified in the 5’ prime UTR regulatory region of *AHCYL2.* Using the position of each variant relative to the position of predicted promoters allowed comparison of the distance to the nearest promotor for each variant. This showed that for each of the six variants identified in the 5 prime UTR regulatory region of *AHCYL2* had different distances to the nearest predicted promoters and the position of promoters differed significantly between the reference and alternative sequences. Further, in the alternative sequence 80 promoters were predicted compared to 56 in the reference suggesting that there may be a difference in the expression of this gene.
